# Long-Term Outcomes of Stereotactic Radiosurgery for Vestibular Schwannoma Associated with Neurofibromatosis Type 2 in Comparison to Sporadic Schwannoma

**DOI:** 10.3390/cancers11101498

**Published:** 2019-10-07

**Authors:** Yuki Shinya, Hirotaka Hasegawa, Masahiro Shin, Takehiro Sugiyama, Mariko Kawashima, Wataru Takahashi, Shinichi Iwasaki, Akinori Kashio, Hirofumi Nakatomi, Nobuhito Saito

**Affiliations:** 1 Department of Neurosurgery, The University of Tokyo Hospital, Tokyo 113-8655, Japan; yukishinya6155@gmail.com (Y.S.); SHIN-NSU@h.u-tokyo.ac.jp (M.S.); mrkawashima-tky@umin.ac.jp (M.K.); hnakatomi-tky@umin.ac.jp (H.N.); nsaito-tky@umin.net (N.S.); 2 Department of Neurologic Surgery, Mayo Clinic, MN 55905, USA; 3 Diabetes and Metabolism Information Center, Research Institute, National Center for Global Health and Medicine, Tokyo 162-8655, Japan; takehiro.sugiyama@gmail.com; 4 Department of Health Services Research, Faculty of Medicine, University of Tsukuba, Ibaraki 305-8575, Japan; 5 Department of Public Health/Health Policy, Graduate School of Medicine, The University of Tokyo, Tokyo 113-8654, Japan; 6 Department of Radiology, The University of Tokyo Hospital, Tokyo 113-8655, Japan; wataru.harry1@gmail.com; 7 Department of Otorhinolaryngology, The University of Tokyo Hospital, Tokyo 113-8655, Japan; iwasaki-oto@h.u-tokyo.ac.jp (S.I.); kashioa-oto@h.u-tokyo.ac.jp (A.K.)

**Keywords:** gamma knife radiosurgery, neurofibromatosis type 2, propensity score matching, stereotactic radiosurgery, tumor control, vestibular schwannoma

## Abstract

The efficacy of radiosurgery for neurofibromatosis type 2 (NF2)-associated vestibular schwannoma (VS) remains debatable. We retrospectively analyzed radiosurgical outcomes for NF2-associated VS compared to sporadic VS using our database of 422 consecutive VS patients. Twenty-five patients with 30 NF2-associated VSs with a mean follow-up of 121 months were identified. NF2-associated VSs exhibited excellent tumor control (10-year cumulative rate, 92% vs. 92% in sporadic VSs; *p* = 0.945) and worse overall survival (73% vs. 97%; *p* = 0.005), mainly due to tumor progression other than the treated VSs. The presence of NF2 was not associated with failed tumor control via multivariate Cox proportional hazard analyses. No difference in radiation-induced adverse events (RAEs) was confirmed between cohorts, and prescription dose (hazard ratio 8.30, 95% confidence interval 3.19–21.62, *p* < 0.001) was confirmed as a risk for cranial nerve injuries via multivariate analysis. Further analysis after propensity score matching using age, volume, and sex as covariates showed that NF2-associated VSs exhibited excellent local control (100% vs. 93%; *p* = 0.240) and worse overall survival (67% vs. 100%; *p* = 0.002) with no significant difference in RAEs. Excellent long-term tumor control and minimal invasiveness may make radiosurgery a favorable therapeutic option for NF2 patients with small to medium VS, preferably with non-functional hearing or deafness in combination with postoperative tumor growth or progressive non-operated tumors, or with functional hearing by patients’ wish.

## 1. Introduction

Neurofibromatosis type 2 (NF2) is a rare autosomal dominant genetic disorder caused by inactivating alterations in the *NF2* gene on chromosome 22q12.2, with a prevalence of around 1 in 60,000 [1,2,3,4]. Patients with NF2 develop multiple tumors in the nervous system, and NF2-associated tumors often contribute to earlier-than-expected death [5]. In particular, bilateral vestibular schwannomas (VS) are the most pathognomonic and diagnostic [6,7]. VSs are also the most common cause of morbidity, potentially resulting in bilateral sensorineural hearing loss, tinnitus, balance difficulty, and ultimately deafness, facial nerve weakness, and possible brainstem compression [8,9]. Classically, two different phenotypes of NF2 are recognized: Wishart type, referring to the more severe phenotype where the affected patient develops multiple tumors at an early age with rapid tumor progression; and the Feiling–Gardner type, referring to a milder form in which the affected patient develops slow-glowing or relatively stable bilateral VSs later in life [10].

VSs also develop sporadically, and treatment options include surgical removal, radiotherapy, and observation. In particular, stereotactic radiosurgery (SRS) is a main therapeutic modality for small to medium-large sporadic VS, offering advantages such as excellent tumor control, low toxicity for the facial nerve, and minimal invasiveness [11,12,13,14,15,16]. Nevertheless, robust evidence regarding the use of SRS for NF2-associated VSs is lacking, and long-term outcomes have not been fully elucidated [17,18,19,20,21,22,23,24,25,26,27]. To address these deficiencies, we conducted the present retrospective study to investigate radiosurgical outcomes for NF2-associated VSs and to compare the results with those for sporadic VS using matched cohort analysis.

## 2. Results

### 2.1. Baseline Characteristics of the Entire Cohort

Patient characteristics are shown in Table 1. Five patients in the NF2 cohort underwent SRS for bilateral tumors at different times. Patients with NF2-associated VS were classified into 11 (37%) with the Wishart type and 19 (63%) with the Feiling–Gardner type. For patients who underwent surgery, the mean ± standard deviation age at time of surgery was 31.6 ± 12.9 years. Patients with NF2-associated VS were significantly younger, more likely to have a history of prior surgery, and showed larger diameter of the VS. Prior to SRS, two patients had a history of radiotherapy for other intracranial lesions that were completely isolated from the VS. Individual characteristics of patients with NF2 are listed in Supplemental Table.

### 2.2. Endpoints for the Entire Cohort

**Figure 1:**
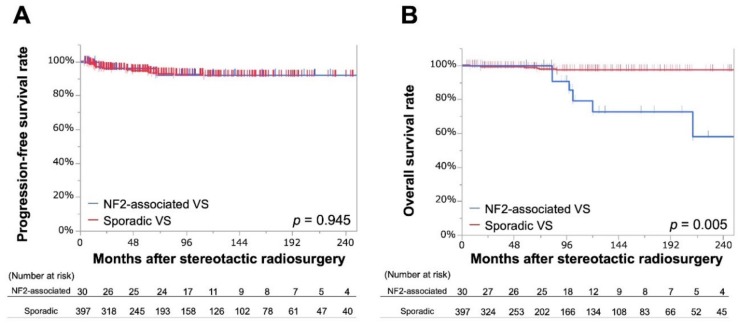
Kaplan–Meier curves for the progression-free survival rate (**A**) and overall survival rate (**B**) before matching comparing neurofibromatosis type 2-associated vestibular schwannoma with sporadic vestibular schwannoma. NF2 = neurofibromatosis type 2; OS = overall survival rate; PFR = progression-free rate; VS = vestibular schwannoma.

In the entire cohort, tumor progression was confirmed in 2 tumors from the NF2 cohort (6.7%) and 24 tumors from the sporadic cohort (6.0%); representing progression-free rates (PFRs) of 96% and 95% at 5 years and 92% and 92% at 10–20 years, respectively. No significant differences were apparent between the two Kaplan–Meier curves (*p* = 0.945; Figure 1A). Prior surgical intervention (hazard ratio [HR] 2.39, 95% confidence interval [CI] 1.07–5.17, *p* = 0.035) was significantly associated with tumor progression in bivariate analyses, but no significant factors were identified from multivariate analysis (Table 2).

Over the entire observation period, radiation-induced adverse events (RAEs) were observed in 4 (13.3%) and 35 (8.8%) patients (including 2 (6.7%) and 23 (5.8%) patients with Common Terminology Criteria for Adverse Events (CTCAE) grade 3–4 RAEs) in the NF2 and sporadic cohorts, respectively (Table 3). No significant differences in rates of any RAEs were identified between cohorts. Prescribed dose >13 gray (Gy) (odds ratio [OR] 7.61, 95%CI 3.00–19.32, *p* < 0.001) and central dose >26 Gy (OR 2.44, 95%CI 1.07–5.57, *p* = 0.034) were significantly associated with cranial nerve injuries according to bivariate analysis, with prescribed dose (OR 8.30, 95%CI 3.19–21.62, *p* < 0.001) also confirmed as significant via multivariate analysis (Table 4).

Before SRS, 8 and 173 patients had serviceable hearing (pure tone average <50 decibel [dB]); over the entire observation period, serviceable hearing was preserved in 4 (50%) and 87 (50%) patients in the NF2 and sporadic cohorts, respectively (Table S2). There was no significant difference in hearing preservation rate between two groups (OR 0.99, 95%CI 0.24–4.08, *p* = 0.987)

Seven (23.3%) and 9 (2.3%) mortalities were observed in the NF2 and sporadic cohorts, respectively, yielding overall survival rates (OS)s of 73% at 10 years and 58% at 20 years in the NF2 cohort and 97% at 10–20 years in the sporadic cohort. Of note, two mortalities due to malignant transformation were observed in sporadic VSs, one of which was previously reported in the literature [28]. A significant difference was seen between the two Kaplan–Meier curves (*p* = 0.005; Figure 1B). No mortalities observed in the NF2 cohort were associated with VS, with progression of intracranial tumor in two cases (at 83 and 212 months), suffocation in two (83 and 275 months), fatal subarachnoid hemorrhage in one (at 102 months), lung tumor in one (at 98 months), and unknown cause in one (at 120 months).

### 2.3. Propensity Score Matching and Patient Background in the Matched Cohort

A total of 30 NF2-associated VSs and 397 sporadic VSs were eligible for matching, which was performed by investigators blinded to treatment outcome. The matching process resulted in a final cohort of 66 VSs eligible for further analyses, including 22 NF2-associated VSs with a mean (± standard deviation) observation period of 122 ± 84 months and 44 sporadic VSs with a mean observation period of 116 ± 101 months (Table 5). During the with-replacement matching process, four sporadic VSs were selected twice and one sporadic VS was matched three times. Three (14%) and 19 (86%) tumors were presumed to be Wishart and Feiling–Gardner types, respectively.

### 2.4. Endpoints in the Matched Cohort

**Figure 2:**
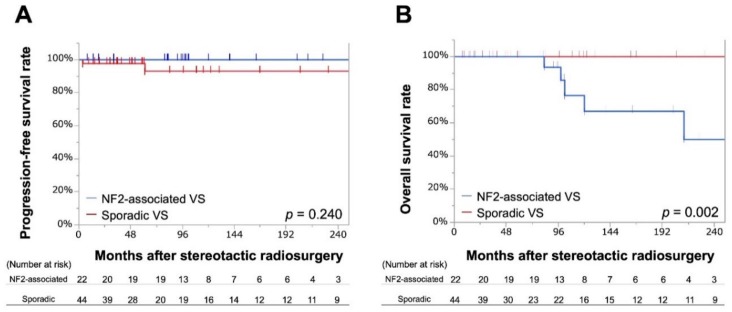
Kaplan-Meier curve for progression-free survival rate (**A**) and overall survival rate (**B**) among the propensity score-matched cohort comparing neurofibromatosis type 2-associated vestibular schwannoma with sporadic vestibular schwannoma. NF2 = neurofibromatosis type 2; OS = overall survival rate; PFR = progression-free rate; VS = vestibular schwannoma.

Among NF2-associated VSs, no tumor showed progression over the observation period after SRS, yielding 100% PFR at 10–20 years; whereas among sporadic VSs, 2 (4.5%) tumors showed progression, yielding PFR of 93% at 10–20 years (Figure 2A).

Over the follow-up period, 3 (13.6%) and 6 (13.6%) RAEs were observed, including 2 (9.1%) and 3 (6.8%) CTCAE grade 3–4 RAEs, 1 (4.5%) and 3 (6.8%) trigeminal neuralgias, 1 (4.5%) and 3 (6.8%) facial nerve palsies, and 2 (9.1%) and 2 (4.5%) hydrocephaluses, respectively, in NF2-associated and sporadic VSs. No significant difference between groups was confirmed for any complications.

Before SRS, 4 and 20 patients had serviceable hearing; over the entire observation period, serviceable hearing was preserved in 2 (50%) and 13 (65%) patients in the NF2 and sporadic cohorts, respectively (Table S3). There was no significant difference in the hearing preservation rate between two groups (OR 0.54, 95%CI 0.06–4.69, *p* = 0.572)

Five and no mortalities were observed over the entire observation period in NF2-associated and sporadic VSs, respectively. OSs were 67% at 10 years and 50% at 20 years for NF2-associated VSs and 100% at 10–20 years for sporadic VSs (*p* = 0.002; Figure 2B).

## 3. Discussion

The present study showed excellent PFR (92% at 10–20 years) for NF2-associated VSs, even though we had used relatively low radiosurgical doses (mean prescribed dose, 13 Gy), with these results bolstered by our long-term observation period (mean, 121 months). OS was significantly lower for the NF2-associated VSs cohort, but this was considered predominantly attributable to causes other than the VS. Overall, presence of NF2 was not associated with radiosurgical outcomes in either multivariate analysis of the entire cohort or matched cohort analysis.

To date, studies focusing on SRS outcomes of NF2-associated VSs have been both limited in number and somewhat controversial (Table S1) [17,19,20,22,23,24,29]. Most importantly, the rate of successful tumor control has varied from study to study, ranging from 41% to 87% at 8–10 years. Likewise, while some earlier studies reported that outcomes of NF2-associated VSs were inferior to those of sporadic VSs [19,20,22,23,29]. the two most recent studies found no difference [17,24]. Such discrepancies might be due to various reasons. First, definitions of successful tumor control differed, with some studies using “tumor control rate”, where a tumor is considered “controlled” if the patient requires no further surgical or radiosurgical intervention after initial SRS, regardless of any increase in tumor size [30,31], while others used PFR [17,20,23,29]. The present study used PFR, as a more sensitive measure for assessing tumor response to SRS.

Second, each institution has its own philosophy of radiosurgical planning in terms of dose and tumor coverage. Optimal radiosurgical doses for NF2-associated VS have not been determined, although lowered doses (<14 Gy) have generally been advocated for sporadic VS since the early 2000s to avoid RAEs [14,32,33]. Indeed, our study findings supported the contention that a dose >13 Gy significantly increases the risk of cranial nerve complications. While higher-dose SRS was recommended in one previous study, the higher complication rate they reported may not encourage this perspective [17]. On the other hand, tumor coverage was not discussed in previous studies, although one study reported a mean target coverage of 91.5% [29]. Although we did not calculate tumor coverage, our policy is to place multiple shots to meticulously circumscribe all tumor contours identifiable as high-intensity areas on contrast-enhanced T1-weighted magnetic resonance imaging (MRI). This should theoretically result in a coverage rate slightly above 100%, although this would be acceptable given our low complication rates. In short, low-dose SRS with sufficient tumor coverage may offer an optimal approach to balancing tumor control and RAEs.

Significant heterogeneity in terms of disease severity exists even within the same disease entity [34,35] and tumor phenotype may affect tumor control [23,24]. Another study reported younger age as a risk factor for failed tumor control, which may represent a rephrasing of this same notion, given that the Wishart type refers to an aggressive phenotype occurring in younger individuals. In the present study, 2 of 14 tumors with Wishart-type NF2 showed failed control, whereas none of the 16 Feiling–Gardner-type tumors showed progression. Although we did not observe any significant difference between the two types, this does not exclude the above-mentioned possibility that tumor phenotype affects the outcomes of SRS. Meanwhile, it should be kept in mind that NF2 patients do not always clearly fall into these two subtypes, as the severity of disease seems to be strongly associated with the pattern of gene mutation, which varies individually and thus an intermediate type may be considered to exist [34,35,36]. Given that genome study has recently been becoming widely available, further study is desirable to see the association between radiosurgical outcomes and mutation subtypes.

Surgical treatment is a standard option for NF2-associated VS and provides immediate significant decompression [5,37,38]. On the other hand, NF2 patients are undeniably likely to need multiple interventions for multiple tumors within the compass of their lifetime, possibly resulting in unexpected increases in physical and psychological hardship. In this regard, SRS could provide a potent treatment option given the favorable outcomes coupled with the minimal invasiveness. In the meantime, as previously reported, SRS is not as effective for large VSs as for small to medium-sized tumors [24]. Surgical resection would thus represent a reasonable option for such cases. Although a previous study shows that surgery may decrease the postoperative tumor growth rate [39], our study design is not adequate to see this effect, as our study did not simply compare VSs without vs. with prior surgery, but compared VSs without prior surgery vs. with prior surgery and “growing trend”.

Our policy has been to generally avoid SRS for patients with preserved hearing. We thus failed to have data enough to address hearing outcomes. Moreover, we do not have thorough data on speech discrimination score for every patient, and thus we alternatively used “50 dB or less in pure tone average” as post-SRS serviceable hearing. This might have overestimated the rate of hearing preservation. However, as far as our analyses went, there was no substantial difference between NF2-associated and sporadic VSs. The current evidence suggests that post-SRS hearing preservation rates for NF2-associated VSs are 33%–93% at 5 years and 44% at 10 years [17,18,20,23,24,29] suggesting that long-term preservation of hearing function is not promising. Given the bilateral presence of VSs, upfront SRS for tumors on the side of preserved hearing may not be recommended. Instead, we would first offer close observation and perform SRS at some point in a timely manner before the tumor becomes too large for SRS. On the other hand, proactively considering SRS for small to medium-sized tumors without serviceable hearing would be acceptable.

Previous studies suggested that vascular endothelial growth factor (VEGF) produced by schwannoma cells might activate tumor growth, leading to associated hearing loss in NF2 patients [40,41], and that bevacizumab, a humanized monoclonal antibody against VEGF, might inhibit this process, bringing hearing improvement and reduction in tumor size [41,42,43,44]. Although it seems very promising, given that bevacizumab is not effective for all cases and its long-term outcomes are yet to be determined, SRS would retain a significant therapeutic value.

This study has several limitations that should be considered when interpreting the results. First, the retrospective nature of the study may have impaired its significance. Given the scarcity of NF2, however, prospective studies are not realistic, and the present method would be the best available option. Second, although we assessed the efficacy of SRS by comparing NF2-associated VSs to sporadic VSs, we would have to also compare them to those who have NF2-associated VSs but were untreated to see the real benefit of SRS. However, leaving them untreated is unethical and hard to justify, so we used the present study design. Third, a significant difference in patient number existed between NF2-associated and sporadic VSs, which might have affected statistical analyses between the two cohorts. To supplement this, we added matched-cohort analysis to reconfirm the consistency of results. In addition, even though we created a matched cohort using propensity score analysis, significant differences in some other variables might have gone unrecognized. Essentially, those limitations could be resolved in further research enrolling more cases with a longer follow-up period. Forth, even though we did not experience any malignant transformation in NF2-associated VSs and the risk is deemed quite low [28,45,46], it is a non-negligible risk of radiation and needs to be fully explained to patients before treatment. Finally, SRS for VSs may also affect the surrounding areas in the posterior fossa including the brainstem, cranial nerves, and vessels. Multiple other tumors may develop as time passes in this area, which may require surgical intervention in the long run. Although it is difficult to measure the radiation effect to the normal anatomies as well as tumors that will arise in the future, radiation may cause some undesirable changes including adhesion and inflammation, which may affect surgical risk. We have to decide treatment strategies with all of them in mind.

## 4. Materials and Methods

### 4.1. Study Population

We retrospectively collected data on consecutive 456 patients with VS who were treated with SRS at our institution between 1990 and 2018. After excluding 34 patients for whom appropriate follow-up data (<3 months) were lacking, 422 patients with 427 VSs including 25 NF2-associated patients with 30 VSs were included in the study. All aspects of the study were approved by the institutional review board of our institution (#2231), and all patients provided written informed consent for study participation.

### 4.2. Radiosurgical Techniques and Post-SRS Management

After head fixation using a Leksell frame (Elekta Instruments, Stockholm, Sweden), stereotactic imaging (computed tomography before July 1996; MRI thereafter) was performed to obtain precise data on the shape, volume, and three-dimensional coordinates of the tumors. Radiosurgical planning was performed by dedicated neurosurgeons and radiation oncologists using commercially available software (KULA planning system until 1998; Leksell Gamma Plan thereafter [Elekta Instruments]. All treatment strategies were discussed at neurosurgical conferences. In principle, treatment was not considered for small asymptomatic tumors in patients with good hearing function. Patients < 70 years old with progressive tumor or post-surgical recurrence were considered good candidates for SRS. Surgery was generally preferred for large tumors (>3 cm in maximum diameter) regardless of age and for medium-sized to large tumors in young patients. As our philosophy, upfront SRS for a NF2 patient on the side of serviceable hearing (Gardner–Robertson hearing classification I and II) was avoided out of concern for the risk of bilateral hearing loss. When a VS on one side clearly showed progression in the setting of bilateral serviceable hearing and the patient had clear desire to proceed with treatment, we considered SRS on the growing side.

Patients were followed-up regularly every 6 months with MRI for the first 3 years, then every 1 year thereafter. The resulting images were evaluated by neurosurgeons and radiologists. Transient expansion, which occurs due to radiation-induced tumor swelling at around 6 months followed by shrinkage at around 18 months with an average peak expansion rate of 47% in volume [47,48] was meticulously distinguished from true tumor progression by evaluating results of consecutive MRI [24]. The clinical conditions of patients as well as responses to treatment were prospectively collected at the time of the hospital visit, whereas data for patients who dropped out of regular follow-up were collected by telephone. Follow-up radiologic images were subsequently gathered by the patient or their attending physicians.

### 4.3. Statistical Analysis

First, baseline characteristics of patients were summarized. Second, PFR and OS in the NF2 and sporadic cohorts were calculated using Kaplan–Meier methods, and compared between groups with the log-rank test. A tumor was considered “progression-free” when it did not show enlargement of >20% in volume on two or more consecutive post-SRS imaging studies [20,23,49,50,51]. Post-SRS hearing outcomes were summarized based on pure-tone average and compared between NF2 and sporadic cohorts with Fisher’s exact test. RAEs were graded according to CTCAE version 4.0 and compared between cohorts with Pearson’s chi-square test. PFR, OS, and RAE rates were evaluated as per tumor. Factors associated with PFR were examined with bi- and multivariate Cox proportional analyses. Factors associated with occurrence of RAEs were examined with logistic regression analysis. Continuous variables (radiosurgical doses, target volume and age) were entered into models after being dichotomized using mean values. Multivariate analyses were performed using presence of NF2, age at SRS, tumor volume, prescription dose, and prior surgical history.

Since significant differences in background characteristics and numbers of patients were expected between NF2-associated and sporadic VSs, we performed propensity score-matching to generate comparable groups for investigating differences in prognosis by the presence of NF2 adjusted for possible confounders. Propensity-score matching with replacement was conducted using the following variables: age, age^2^ (quadratic term), age^3^ (cubic term), volume, volume^2^ (quadratic term), volume^3^ (cubic term), sex (categorical), and age × sex (interaction term), with a matching ratio (NF2-associated VS: sporadic VS) of 1:2 and a caliper distance of 0.03. For matched groups, we conducted the same bivariate analyses for PFR, RAEs and OS. All statistical analyses were performed using JMP Pro 14 software (SAS Institute Inc., Cary, NC, USA) and Stata 15.1 (StataCorp, College Station, TX, USA).

## 5. Conclusions

We confirmed a lack of significant difference in outcomes of SRS between NF2-associated and sporadic VSs. The excellent long-term tumor control (92% at 10–20 years) and low complication rate (CTCAE grade 3–4 RAEs, 6.7%) support the use of SRS as a favorable therapeutic option first-line treatment for NF2 patients with small to medium VS, preferably with non-functional hearing or deafness in combination with postoperative tumor growth or progressive non-operated tumors, or with functional hearing by patients’ wish. Needless to say, determining a comprehensive strategy is of central importance not only for VSs, but also for all other intracranial tumors, with due consideration of the individual background as well as the systemic condition.

## Figures and Tables

**Table 1:** Baseline characteristics and dosimetry data of patients before matching. * Values of *p* < 0.05 are considered statistically significant. NF2 = neurofibromatosis type 2; VS = vestibular schwannoma; SRS = stereotactic radiosurgery; SD = standard deviation; Gy = gray.

Variables	NF2-Associated VSs	Sporadic VSs	*p* Value
Number of patients (tumors), n	25 (30)	397 (397)	/
Age at SRS, year, mean ± SD	38 ± 18	56 ± 13	<0.001 *
Observation period, months, mean ± SD	121 ± 82	103 ± 91	0.300
Target volume, cm^3^, mean ± SD	4.4 ± 4.1	2.0 ± 2.0	<0.001 *
Prescription dose, Gy, mean ± SD	13 ± 0.4	13 ± 0.1	0.883
Central dose, Gy, mean ± SD	27 ± 0.7	27 ± 0.2	0.760
Male sex, *n* (%)	6 (20.0)	196 (49)	0.002 *
Prior surgical intervention, *n* (%)	19 (63)	88 (22)	<0.001 *

**Table 2:** Results of bi- and multivariate analyses of tumor progression before matching. * Values of *p* < 0.05 are considered statistically significant. HR = hazard ratio; CI = confidence interval; NF2 = neurofibromatosis type 2; SRS = stereotactic radiosurgery; Gy = gray.

Variables	Bivariate		Multivariate	
	*p* Value	HR (95%CI)	*p* Value	HR (95%CI)
Presence of NF2 (vs. absence)	0.945	0.95 (0.15–3.21)	0.424	0.56 (0.09–2.09)
Age at SRS >55 years (vs. ≤55 years)	0.270	0.65 (0.29–1.40)	0.440	0.72 (0.31–1.65)
Volume >2.0 cm^3^ (vs. ≤2.0 cm^3^)	0.449	1.35 (0.61–2.93)	0.503	1.32 (0.58–2.91)
Prescription dose >13 Gy (vs. ≤13 Gy)	0.999	1.00 (0.44–2.18)	0.805	0.90 (0.39–2.01)
Central dose >26 Gy (vs. ≤26 Gy)	0.640	0.83 (0.38–1.80)	/	/
Male sex (vs. female sex)	0.072	0.48 (0.20–1.07)	/	/
Prior surgery (vs. absence of prior surgery)	0.035 *	2.39 (1.07–5.17)	0.051	2.31 (1.00–5.18)

**Table 3:** Summary of radiation-related adverse events before matching. * Values of *p* < 0.05 are considered statistically significant. NF2 = neurofibromatosis type 2; VS = vestibular schwannoma; CTCAE = Common Terminology Criteria for Adverse Events version 4.0.

Variables	NF2-associated VSs	Sporadic VSs	*p* Value
All complications	4 (13.3)	35 (8.9)	0.69
-Trigeminal neuralgia	1 (3.3)	11 (2.8)	0.86
-Facial paresis	2 (6.7)	18 (4.5)	0.59
-Hydrocephalus	2 (6.7)	15 (3.8)	0.44
-CTCAE gr. 3–4	2 (6.7)	23 (5.8)	0.84

**Table 4:** Results of bi- and multivariate analyses of cranial nerve injuries as radiation-related adverse events before matching. * Values of *p* < 0.05 are considered statistically significant. OR = odds ratio; CI = confidence interval; NF2 = neurofibromatosis type 2; SRS = stereotactic radiosurgery. Gy = gray.

Variables	Bivariate		Multivariate	
	*p* Value	OR (95%CI)	*p* Value	OR (95%CI)
Presence of NF2 (vs. absence)	0.937	1.06 (0.24–4.72)	0.832	0.84 (0.17–4.20)
Age at SRS >55 years (vs. ≤55 years)	0.712	0.86 (0.40–1.88)	0.432	1.41 (0.60–3.32)
Volume >2.0 cm^3^ (vs. ≤2.0 cm^3^)	0.425	1.38 (0.63–3.02)	0.496	1.34 (0.57–3.15)
Prescription dose >13 Gy (vs. ≤13 Gy)	<0.001 *	7.61 (3.00–19.32)	<0.001 *	8.30 (3.19–21.62)
Central dose >26 Gy (vs. ≤26 Gy)	0.034 *	2.44 (1.07–5.57)	/	/
Male sex (vs. female sex)	0.063	0.45 (0.19–1.05)	/	/
Prior surgery (vs. absence of prior surgery)	0.891	1.06 (0.44–2.60)	0.915	1.05 (0.40–2.76)

**Table 5:** Parameters used for propensity score matching and the percentage bias between cohorts. * Values of *p* < 0.05 are considered statistically significant. NF2 = neurofibromatosis type 2; VS = vestibular schwannoma; %bias = percentage bias; SD = standard deviation.

Variables	NF2-Associated VS (*n* = 22)	Sporadic VS (*n* = 44)	*p* Value	%Bias
Volume, cm^3^, mean ± SD	3.2 ± 0.4	3.1 ± 0.3	0.827	3.4
Age at SRS, year, mean ± SD	45 ± 15	46 ± 16	0.937	−2.1
Male sex, *n* (%)	6 (27)	12 (27)	1.000	0.0

## Supplementary Materials

The following are available online at https://www.mdpi.com/2072-6694/11/10/1498/s1, Table S1: Individual characteristics of patients with NF2 before matching. CNS = central nervous system; F-G = the Feiling–Gardner type; HS = hypoglossal schwannoma; JS = jugular foramen schwannoma; MGM = meningioma; NF2 = neurofibromatosis type 2; OS = oculomotor schwannoma; other S = other schwannoma; SS = spinal schwannoma; TS = trigeminal schwannoma; VS = vestibular schwannoma; W = the Wishart type. Table S2. Hearing results on the basis of pre-SRS PTA before matching. SRS = stereotactic radiosurgery; PTA = pure-tone average; NF2 = neurofibromatosis type 2; VS = vestibular schwannoma. Table S3. Hearing results on the basis of pre-SRS PTA in the propensity score-matched cohort.
