# Supplementary material for: Long-Term Outcomes of Stereotactic Radiosurgery for Vestibular Schwannoma Associated with Neurofibromatosis Type 2 in Comparison to Sporadic Schwannoma
Source: Cancers (Basel). 2019 Oct 7;11(10):1498. doi: 10.3390/cancers11101498 (PMC6827030; doi:10.3390/cancers11101498)
Supplement: Supplementary file 1 [file cancers-11-01498-s001.pdf]

Supplementary Materials: Long-Term Outcomes of Stereotactic Radiosurgery for Vestibular Schwannoma Associated with Neurofibromatosis Type 2 in Comparison to Sporadic Schwannoma

Yuki Shinya, Hirotaka Hasegawa, Masahiro Shin, Takehiro Sugiyama, Mariko Kawashima, Wataru Takahashi, Shinichi Iwasaki, Akinori Kashio, Hirofumi Nakatomi and Nobuhito Saito

Table S1. Individual characteristics of patients with NF2 before matching. CNS = central nervous system; F-G = the Feiling-Gardner type; HS = hypoglossal schwannoma; JS = jugular foramen schwannoma; MGM = meningioma; NF2 = neurofibromatosis type 2; OS = oculomotor schwannoma; other S = other schwannoma; SS = spinal schwannoma; TS = trigeminal schwannoma; VS = vestibular schwannoma; W = the Wishart type.

	**Phenotype**	**Age at Diagnosis** (years)	**Age at GKS** (years)	**Unilateral or Bilateral** VS	**CNS Tumors**
1-1<br>1-2	F–G	22	22<br>33	Bilateral	MGM,TS,SS,Other S
2	F–G	54	54	Bilateral	OS, TS
3	F–G	58	58	Bilateral	None
4	F–G	33	39	Bilateral	MGM, Other S
5	F–G	21	27	Bilateral	MGM, TS, HS, SS, Other S
6-1<br>6-2	W	13<br>13	13<br>29	Bilateral	MGM, OS, TS, Other S
7	W	18	18	Single	Other S
8-1<br>8-2	F–G	37	39<br>50	Bilateral	None
9	F–G	37	44	Bilateral	MGM, TS, JS
10	F–G	49	61	Bilateral	MGM
11	F–G	46	69	Single	MGM, Other S
12	F–G	46	60	Single	MGM, TS, JS, SS
13-1<br>13-2	W	19	19<br>23	Bilateral	MGM, TS, JS, SS, Other S
14-1<br>14-2	W	7	16<br>21	Bilateral	MGM, SS, Other S
15	F–G	28	32	Bilateral	OS, TS, SS, Other S
16	W	17	36	Single	HS, SS
17	W	11	20	Bilateral	MGM, OS, TS, JS, HS, SS, Other S
18	W	15	27	Bilateral	OS, TS, JS, SS
19	F–G	72	72	Bilateral	None
20	F–G	32	43	Bilateral	SS
21	F–G	55	71	Bilateral	MGM, SS
22	W	17	20	Bilateral	SS
23	F–G	25	46	Bilateral	SS
24	F–G	29	37	Bilateral	MGM, TS, JS, SS, Other S
25	F–G	34	49	Bilateral	SS

Table S2. Hearing results on the basis of pre-SRS PTA before matching. SRS = stereotactic radiosurgery; PTA = pure-tone average; NF2 = neurofibromatosis type 2; VS = vestibular schwannoma.

**Post-SRS PTA, *n* (%)**	**Pre-SRS PTA**			
	NF2-associated VS		Sporadic VS	
	<31 dB (*n* = 4)	31–50 dB (*n* = 4)	<31 dB (*n* = 96)	31–50 dB (*n* = 77)
<31 dB	1 (25)	0 (0.0)	35 (37)	1 (1)
31–50 dB	1 (25)	2 (50)	23 (24)	28 (36)
Serviceable		4 (50)		87 (50)
51–90 dB	1 (25)	1 (25)	33 (34)	38 (49)
>91 dB	1 (25)	1 (25)	5 (5)	10 (13)
Non-serviceable		4 (50)		86 (50)

Table S3. Hearing results on the basis of pre-SRS PTA in the propensity score-matched cohort. SRS = stereotactic radiosurgery; PTA = pure-tone average; NF2 = neurofibromatosis type 2; VS = vestibular schwannoma.

**Post-SRS PTA, *n* (%)**	**Pre-SRS PTA**			
	NF2-associated VS		Sporadic VS	
	<31 dB (*n* = 2)	31–50 dB (*n* = 2)	<31 dB (*n* = 12)	31–50 dB (*n* = 8)
<31 dB	1 (50)	0 (0.0)	4 (33)	0 (0.0)
31–50 dB	0 (0.0)	1 (50)	4 (33)	5 (63)
Serviceable		2 (50)		13 (65)
51–90 dB	1 (50)	0 (0.0)	4 (33)	3 (38)
>91 dB	0 (0.0)	1 (50)	0 (0)	0 (0)
Non-serviceable		2 (50)		7 (35)
